# Measurement Property Evaluation of the Arabic Version of the Patient-Specific Functional Scale for Patients with Stroke

**DOI:** 10.3390/healthcare11111642

**Published:** 2023-06-03

**Authors:** Mohammad A. ALMohiza, Mohammed A. Khafaji, Faisal Asiri, Muhammad O. Al-Heizan, Ali H. Alnahdi, Ravi Shankar Reddy

**Affiliations:** 1 Department of Rehabilitation Sciences, College of Applied Medical Sciences, King Saud University, Riyadh 11362, Saudi Arabia; mmohiza@ksu.edu.sa (M.A.A.); malheizan@ksu.edu.sa (M.O.A.-H.); alialnahdi@ksu.edu.sa (A.H.A.); 2 Department of Rehabilitation, King Abdulaziz Medical City, Riyadh 11426, Saudi Arabia; khafajimo@mngha.med.sa; 3 Department of Medical Rehabilitation Sciences, College of Applied Medical Sciences, King Khalid University, Abha 61421, Saudi Arabia; fasiri@kku.edu.sa

**Keywords:** stroke, outcomes, functional scales, measurement properties

## Abstract

Neurological disorders refer to disorders that occur due to disease or damage to the nervous system. Stroke is one of the most common neurological disorders in which individuals commonly present with motor and sensory deficits, leading to the limitations on the activities of daily life. Outcome measures are used to assess and monitor patients’ condition change. The patient-specific functional scale (PSFS) is an outcome measure used to assess changes in performance levels in participants with a functional disability during daily activities. This study aimed to assess the reliability and validity of the Arabic version of the patient-specific functional scale (PSFS-Ar) in individuals with stroke. A longitudinal cohort study was used to examine the reliability and validity of the PSFS-Ar in patients with stroke. All participants completed the PSFS-Ar in addition to other outcome measures. Fifty-five individuals participated (fifty male, five female). The PSFS-Ar showed excellent test–retest reliability, with ICC2,1 = 0.96, *p* < 0.001. The SEM and MDC95 of the PSFS-Ar were 0.37 and 1.03, respectively. No floor and ceiling effect was observed in this study. Additionally, the construct validity of the PSFS-Ar showed 100% satisfaction with the pre-defined hypotheses. Since the number of female participants was very small in this study, the findings were established for male individuals with stroke. This study showed that the PSFS-Ar is a reliable and valid outcome measure for male individuals with stroke.

## 1. Introduction

Neurological disorders refer to different dysfunctions that occur as a result of damage or disease of the nervous system [1]. It is ranked as the second leading cause of death, behind cardiovascular diseases [2]. Acquired brain injury (ABI) implies damage to the brain tissue that happens after birth, whether that damage is traumatic or non-traumatic [3]. One of the most prevalent types of non-traumatic ABI is stroke, which is characterized by bleeding in the brain or a blockage of blood supply to the brain [3,4,5]. Individuals with stroke commonly present with motor and sensory deficits resulting in hemiparesis or hemiplegia [6,7]. As part of a multidisciplinary team, physical therapists utilize many methods that help individuals with stroke to restore their functional status [8].

Outcome measures are created to assess and monitor the change in the patient’s condition [9]. Additionally, outcome measures provide ideal tools for communication among healthcare providers [9]. The term patient-reported outcome measures (PROMs) falls under the category of outcome measurements in which patients report the changes in their health status without influence from healthcare providers [10]. PROMs aim to assess changes in the health status from the patient’s perspective [11]. On the other hand, patient-centered outcome measures (PCOMs) are outcome measures that facilitate healthcare by focusing on matters that are important to the patient rather than the healthcare providers [12]. The patient-specific functional scale (PSFS) is one of the self-reported outcome measures used to determine functional difficulties [13]. In this measure, the patient will report and rate up to five important activities considered difficult to perform, and the therapist will document them [13,14]. Therefore, the PSFS can be categorized as a PROM and a PCOM [15]. It has been recommended to be used as part of the management of neurological disorders [14,16]. To the best of our knowledge, the psychometric properties of the PSFS have never been evaluated in individuals with stroke. The aim of this study was to assess the reliability and validity of the Arabic version of the patient-specific functional scale (PSFS-Ar) in individuals with stroke.

## 2. Materials and Methods

### 2.1. Study Design

A longitudinal cohort study was used to examine the reliability and validity of the PSFS-Ar in patients with stroke. Ethical approval was obtained from the Institutional Review Board (IRB) committee at King Saud University and King Abdullah International Medical Research Center (KAIMRC). All participants signed a consent form prior to participating in the study.

### 2.2. Setting and Participants

Adult patients from the National Guard Hospital Affair (NGHA) in Riyadh City were recruited. The physical therapists at the neuro-rehabilitation outpatient clinic collected the data. Each therapist received training on the study protocol. Patients with stroke from both genders were included if they were (1) clinically diagnosed as individuals with stroke, (2) between 18 and 85 years old, and (3) Arabic speakers. Patients with stroke were excluded if they (1) showed signs of cognitive impairment or inability to express their functional limitations, (2) had limited physical activity due to conditions other than stroke, (3) had had an upper limb or lower limb fracture in the past three months, (4) had limb amputation, (5) were dependent in ambulation (scored 0 in the Functional Ambulation Categories assessment), and (6) were blind.

Given the team’s busy schedule at the NGHA neuro-rehabilitation outpatient clinic, the regular procedure does not require an application of a standardized cognitive measure unless the patient shows a significant need for special cognitive screening. This need for a standardized cognitive screening is based on simple and structured cognitive questions regarding orientation, attention, communication, and memory. The simple cognitive questions and tasks include the following: (1) Does the patient respond to greetings and are they able to introduce himself/herself? (2) Is the patient aware of the time, date, and location? (3) Is the patient able to explain his/her chief complaint? (4) Give the patients simple instructions (such as standing up from the chair or taking off their jacket) and observe if they can follow these commands. (5) Tell the patients three items (such as school, apple, and car) and have them repeat them immediately, then ask about these items after 3–5 min.

### 2.3. Procedure

Participants completed three self-reported questionnaires: the PSFS-Ar, Stroke-Specific Quality of Life Scale (SSQOL-Ar), and the Global Rating of Change (GROC). Additionally, three observer outcome measures were performed, including The Berg Balance Scale (BBS), Timed Up and Go (TUG), and Functional Ambulation Categories (FAC). In the first session, participants completed the first test (T1) for the test–retest reliability of the PSFS-Ar, which also was used to test the validity. All other outcome measures (except the Global Rating of Change) were completed during the first visit for construct validity. During the second visit, which took place after 4 to 7 days, participants completed the second test (T2) of the PSFS-Ar for test–retest reliability. They were asked to rate the change in their health status by using the GROC in order to determine that the patient’s health status had not changed between (T1) and (T2). For test–retest reliability, the patient’s health status was considered unchanged if they scored from +2 to −2.46 The gap between (T1) and (T2) was set to 4–7 days to ensure that the patient did not recall the scores of the activities in the PSFS-Ar and that his/her health status did not change between (T1) and (T2). Similarly, Alnahdi et al. [17] set the time between (T1) and (T2) to 2–7 days.

#### 2.3.1. The Patient-Specific Functional Scale (PSFS)

The PSFS is a self-reported outcome measure used to measure the level of difficulty encountered by the patient when performing activities of daily life [13,18]. At the initial assessment, the therapist asks the patient to identify three to five important activities that he/she has difficulty with or cannot perform during daily life [13,18]. Then, the patient rates their ability to achieve each activity from 0 to 10, where 0 is unable to complete the activity, and 10 is able to complete it at the same level as before the injury. Then, the therapist asks the patient to rate his/her ability to perform the activity at follow-up sessions to detect changes in the patient’s condition [13,18]. The total score is the average of the scores of activities selected by the patient [13,18]. The PSFS English original version has high test–retest reliability (ICC = 0.97 and SEM = 0.41 rating points) in patients with lower back pain [13]. The PSFS test–retest reliability was observed in acutely hospitalized patients with different conditions, including neurological and musculoskeletal conditions, among elderly patients with and without cognitive impairments with adequate ICC = 0.76 [19]. Moreover, use of the PSFS was feasible in patients with Parkinson’s disease since patients were able to identify their disability [20]. Additionally, the PSFS showed applicability in patients with ABI since most patients were able to complete and identify the activities, they were unable to perform daily, although 8% were unable to complete the scale due to severe cognitive or language impairments [21].

#### 2.3.2. The Berg Balance Scale (BBS)

The BBS is an observer measure for balance in daily activities that contains a 14-item scale [22]. Each of these items is scored from 0 to 4, where 0 represents the inability to complete the task and 4 represents the ability to complete the task independently [22]. The total score is between 0 and 56, with a higher score indicating better balance [23]. Scores of 0 to 20 indicate poor balance, 21 to 40 imply fair balance, and 41 to 56 denote good balance [22]. It was observed that the BBS was an instrument with excellent validity and reliability used to evaluate the balance and functional mobility in the stroke population [24]. The BBS has excellent test–retest reliability (ICC = 0.95) and excellent construct validity, where admission scores of the BBS were correlated with Functional Independence Measure (FIM) admission scores (r = 0.76) in individuals with stroke [25,26].

#### 2.3.3. The Timed Up and Go (TUG) Test

The TUG test is used to measure functional mobility [27]. Participants with or without assistive devices sit on a standardized armchair, stand up, and walk straight for 3 m, then turn around and walk back to sit on the chair [27]. The therapist times the activity by using a stopwatch. Less than 10 s indicates complete independence during functional mobility, less than 20 s indicates independence for most functional mobility, and more than 30 s represents dependency during functional mobility [27]. The TUG has excellent test–retest reliability (ICC = 0.96) in patients with stroke [28]. The TUG has a moderate negative correlation with BBS in patients with chronic stroke [29]. The TUG has excellent convergent validity with BBS (r = 0.70 to 0.83, *p* < 0.001) in patients with stroke [30].

#### 2.3.4. The Functional Ambulation Categories

The FAC is a functional walking test that assesses ambulation ability [31]. It is scored from 0 to 5, where 0 represents non-functional ambulation, 1 indicates ambulation with support from one person, 2 represents ambulation with light touch assistance from one person, 3 denotes ambulation with supervision, 4 indicates ambulation on level surfaces only, and 5 represents walking independently on the level and non-level surfaces [31,32]. The FAC has excellent reliability and good validity in patients with stroke [32]. Moreover, The FAC has very good interrater reliability (K = 0.91), and good concurrent validity with a 6 min walk test (6MWT) (r = 0.795, *p* < 0.001), in patients with stroke [32].

#### 2.3.5. The Stroke-Specific Quality of Life Scale

The SSQOL is a questionnaire used to assess the quality of life of individuals with stroke [32]. It comprises 49 questions covering 12 domains (mobility, upper limb functions, social role, energy, self-care, family role, work-productivity, language, mood, personality, thinking, and vision) [32]. Each question is scored from one to five, with higher scores representing a better quality of life. The unweighted means of associated questions are used to compute each domain score, while the unweighted mean of all domains is utilized to calculate the overall score for the SSQOL [33,34]. The SSQOL has excellent internal consistency (Cronbach’s alpha > 0.73) [32]. The SSQOL has been translated into the Arabic language (SSQOL-Ar) [33]. The Arabic version has good test–retest reliability (ICC = 0.77 to 0.94) and good internal consistency (Cronbach’s alpha = 0.78 to 0.94) [33]. It also has acceptable construct validity (r2 = 0.06 to 0.55) for populations with mild to moderate stroke [33].

#### 2.3.6. The Global Rating of Change Scale

The GROC is a self-report outcome measure that is used to detect the change in a patient’s health status, whether it has improved, deteriorated, or not changed [35,36]. The therapist asks the participant to evaluate the change in his/her health status between the initial assessment and the second visit [35,36]. The GROC scores range between −5 and +5, with (−5) indicating substantially worse, (+5) indicating substantially better, and zero indicating no change [35,36].

### 2.4. Statistical Analyses

Test–retest reliability is usually evaluated during questionnaire development. The intra-class correlation coefficient (ICC) is used most frequently to estimate test–retest reliability [37]. An ICC value equal to or greater than 0.7 is considered an adequate value [37]. An ICC2,1 2-way random-effects model was deemed appropriate for this study to promote the generalizability of the reliable results to other physical therapists [38]. The measurement error of the PSFS-Ar was calculated using the standard error of measurement (SEM) with the following formula: SEM = standard deviation × √(1 − ICC2,1). The scale’s minimal detectable change (MDC) at 95% confidence was determined using the following formula: MDC95 = SEM × 1.96 × √2. According to McHorney et al. [39], the floor and ceiling effect is estimated by calculating the percentage of participants who received the highest and lowest possible score on a scale. If more than 15% of the participants scored the highest or lowest score in the PSFS-Ar, it is considered to have a floor and ceiling effect [39]. The Spearman coefficient was used to evaluate all correlational hypotheses. A value near ±1 reflects a significant positive/negative relationship, while a value close to zero indicates no relationship [40,41]. A correlation coefficient that is less than 0.3 indicates a weak relationship [40,41]. If it is between 0.31 and 0.5, the relationship is moderate, and if it is higher than 0.5, it is strong [40,41]. The construct validity of the PSFS-Ar was sustained when at least 75% of the results correlated with this hypothesis [42]. IBM SPSS Statistics 22 (IBM Corp, Armonk, NY, USA) was used for all analyses. The significant standard was set at 0.05.

## 3. Results

**Figure 1:**
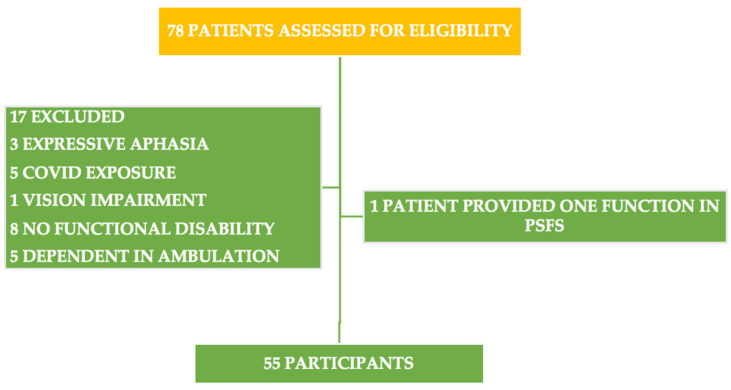
Summary of the participants’ demographic data.

Seventy-eight subjects met the inclusion criteria. One individual was excluded after agreeing to participate because he mentioned only one functional limitation in the PSFS. Three participants with expressive aphasia were excluded because they had limited speech and, thus, could not explain their functional disability in the PSFS. The functional ability of five individuals was decreased after they were exposed to COVID-19. Therefore, they were excluded since their scores on the PSFS-Ar might have been affected. One subject was excluded due to partial visual impairment that affected his balance and walking capability. Eight individuals were excluded since they reported that stroke did not affect their functional ability. Additionally, five subjects were excluded since they depended on ambulation (scored 0 in the Functional Ambulation Categories). Therefore, fifty-five subjects participated in this study (Figure 1).

All participants had good cognitive ability based on the structured questions utilized to screen for cognitive impairment.

Among the participants were fifty male patients (90.9%) and five female patients (9.9%) with a mean age of 62.3 ± 11.3. Fifty-three patients (96.4%) suffered from ischemic stroke, and two patients (3.6%) had hemorrhagic stroke. The clinical staging of stroke was classified into 26 individuals in the subacute (early/late) stage (47.3%) and 29 individuals in the chronic stage (52.7%), while no participants in the hyper-acute or acute stages met the inclusion criteria. The clinical diagnoses were as follows: fifteen participants (27.3%) had basal ganglia stroke, nine patients (16.4%) had corona radiata stroke, three patients (5.5%) had internal capsule stroke, one participant (1.8%) had corpus callosum stroke, five participants (9.1%) had thalamic stroke, eight participants (14.8%) had pontine stroke, one participant (1.8%) had medullary stroke, seven patients (12.7%) had middle cerebral artery (MCA) stroke, five patients (9.1%) had posterior cerebral artery (PCA) stroke, and one patient (1.8%) had anterior cerebral artery (ACA) stroke. Twenty-eight patients (50.9%) ambulated without using an assistive device, thirteen participants (23.6%) walked with a cane, seven patients (12.7%) walked with a hemi-walker frame, four participants (7.3%) walked with a walker frame, and three participants (5.5%) required an assistive device and one-person supervision while walking. The majority of the participants were right-handed (51 patients, 92.7%), and 4 participants (7.3%) were left-handed (Table 1).

Furthermore, there is a brief analysis of the total score of the outcome measures in Table 2.

In total, 55 participants performed the PSFS-Ar test–retest, with an average period of 5 days and a range of 4–12 days between the first and second sessions. Two participants exceeded the limit for the number of days between the test and retest (8 and 12 days); they reported 0 and 2 in GROC, respectively, so they were deemed qualified to be included in the test–retest analysis. However, two participants rated the change in their health status as 3 and 4 based on the GROC, which disqualified them from being included in the test–retest analysis. Therefore, 53 patients reported no change in their health condition in the study and were included in the test–retest analysis. The PSFS-Ar showed excellent test–retest reliability, with ICC2,1 = 0.96, *p* < 0.001 (Table 3 and Table 4).

The MDC95 for the PSFS-Ar was 1.03 (Table 4). The PSFS-Ar (T1) scores were used to explore the floor and ceiling effect. No participants (0%) reached a score of zero, the lowest score, and no participants (0%) scored 10, the highest score. Hence, no floor and ceiling effect of the PSFS-Ar was observed in this study.

**Figure 2:**
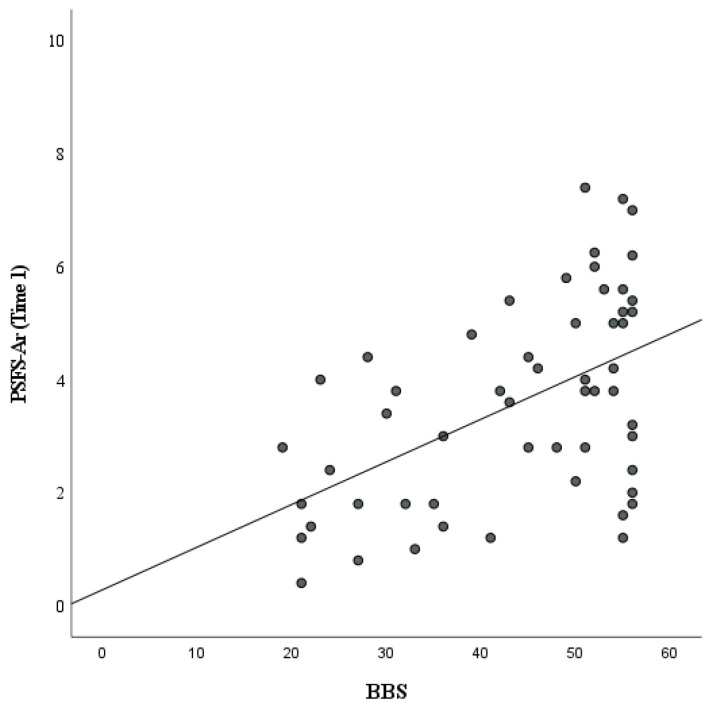
Correlation between Patient-Specific Functional Scale—Arabic (PSFSAr) and Berg Balance Scale (BBS).

**Figure 3:**
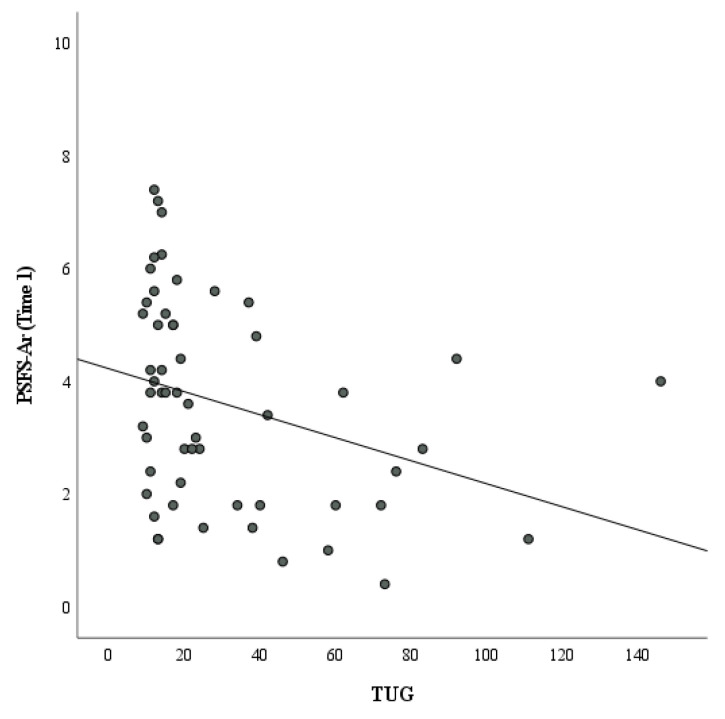
Correlation between Patient-Specific Functional Scale—Arabic (PSFSAr) and Timed Up and Go (TUG).

Data from fifty-five patients qualified for the construct validity analysis. Although their data are continuous, the BBS and TUG data in this study violated the assumptions of parametric statistics. Therefore, the PSFS-Ar showed a significant positive moderate correlation with the BBS (r = 0.45, *p* < 0.001) (Figure 2) and a significant moderate negative correlation with the TUG (r = −0.39, *p* = 0.004) (Figure 3) (Table 5).

**Figure 4:**
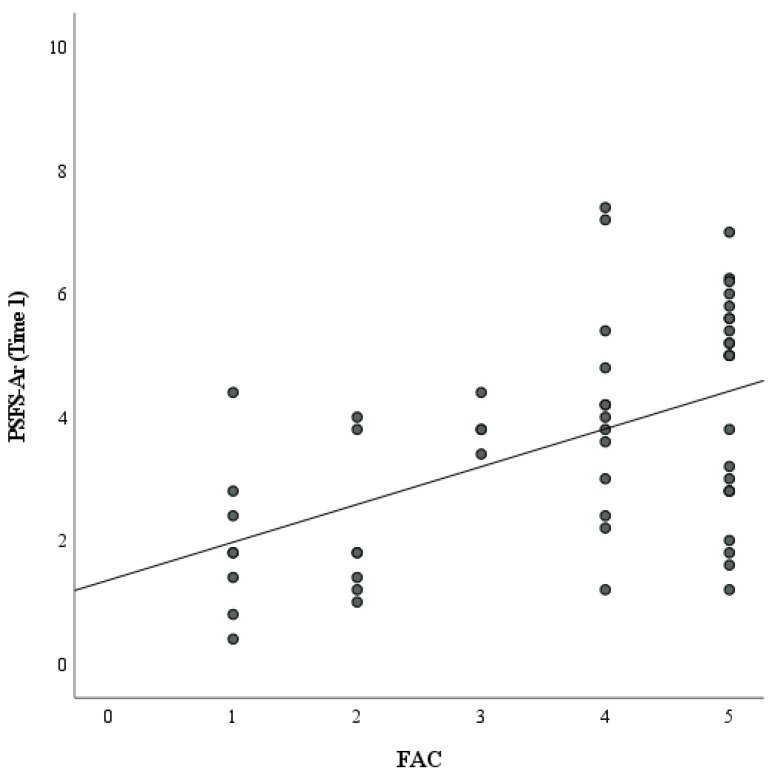
Correlation between Patient-Specific Functional Scale—Arabic (PSFSAr) and Functional Ambulation Categories (FAC).

The FAC and SSQOL-Ar are considered ordinal data. Therefore, the relationship between the PSFS-Ar and these outcome measures was assessed using the Spearman coefficient. There was a significant positive moderate correlation with the FAC scale (r = 0.47, *p* < 0.001) (Figure 4).

Additionally, for the domains in the SSQOL-Ar, there was a significant positive moderate correlation with the mobility domain in the SSQOL-Ar (r = 0.49, *p* < 0.001), a weak correlation with the mood domain in the SSQOL-Ar (r = 0.16, *p* = 0.2), a significant positive moderate correlation with the self-care domain in SSQOL-Ar (r = 0.5, *p* < 0.001), and a significant moderate positive correlation with the upper limb function domain in the SSQOL-Ar (r = 0.43, *p* < 0.001) (Table 5). All the pre-defined hypotheses were met (100%), which supports the construct validity of the PSFS-Ar for individuals with stroke.

## 4. Discussion

The goal of this study was to assess the measurement properties of the PSFS-Ar for Arabic-speaking patients with stroke. Fifty-five participants with stroke completed all outcome measures, with an average age of 62.3 ± 11.3 years.

No patients in the hyper-acute and acute stages met the inclusion criteria in this study since the data were collected in an outpatient setting where most patients are in the subacute (early/late) stages. The PSFS can be more suitable for patients with stroke in the subacute (early/late) and chronic stages than the hyper-acute and acute stages since patients in the latter stages may not yet be aware of their functional limitations [43,44]. In other words, patients with stroke in the hyper-acute and acute stages may not face the hardship of ADLs till they reach the subacute and chronic stages, where they tend to be more independent in their daily activities and can build a better familiarity with their limitations on daily activities. Jorgensen et al. [45] summarized that severity at the onset of stroke plays an important part in functional recovery. Therefore, mild or moderate strokes have a potential recovery within three months, while recovery from more severe strokes takes a longer time [45]. Hankey et al. [46] reported that functional independence improves in the first 6 months after stroke, and it may reach 18 months in some patients, keeping in mind that recovery highly depends on the severity of the stroke. Hurford et al. [47] reported that cognitive impairments are expected in the first month of a stroke incident. However, they decrease within three months. Given the busy schedule of the team at the NGHA neuro-rehabilitation outpatient clinic, the regular practice does not require applying standardized cognitive scales unless the patient clearly shows the need for special cognitive screening based on simple and structured questions regarding orientation, attention, communication, and memory. Based on these cognitive questions, all patients who participated in this study did not require special cognitive screening. Therefore, they were cognitively intact. The cognitive questions used in this study are found in different standardized measures of cognition, such as the MMSE and MOCA [48,49]. Additionally, these cognitive questions are part of the mental status screening of the clinical neurologic examination [50]. Heldmann et al. [47] reported that the PSFS is a valid, reliable, sensitive, and feasible instrument to evaluate functional limitation and progression in elderly patients with or without cognitive impairments [19]. The findings in this study were based on individuals with mild to moderate cognitive impairments. They cannot be generalized to individuals with severe cognitive impairments based on the Mini-Mental State Examination (MMSE) [19]. Heldmann et al. [19] reported that their findings were the same for intact and cognitively impaired individuals and there was a similar average time to complete the PSFS in both groups [19]. While Heldmann et al. [47] evaluated the applicability of the Patient-Specific Functional Scale (PSFS) in elderly patients with mild to moderate cognitive impairments, their study did not specifically focus on cases of agnosia or apraxia resulting from cortical damage. Diagnosing agnosia or apraxia involves assessing specific deficits in sensory perception or motor planning, which may require specialized assessments beyond the scope of the PSFS. Additionally, their study acknowledges that the findings cannot be generalized to individuals with severe cognitive impairments as determined using the Mini-Mental State Examination (MMSE). While the PSFS showed comparable results between cognitively intact individuals and those with impairments, the manuscript does not address the scoring issues related to agnosia or apraxia. Further research is needed to explore the use of the PSFS in evaluating functional limitation and progression, specifically in individuals affected by agnosia or apraxia.

Test–retest reliability refers to the consistency of measurement data when repeated over time [51]. The ICC2,1 (two-way random model for agreement) was used to explore the test–retest reliability for the PSFS-Ar. An ICC2,1 score less than 0.5 indicates poor reliability, a score of 0.5 to 0.75 represents moderate reliability, a score of 0.75 to 0.9 means good reliability, and a score greater than 0.90 reflects excellent reliability [38]. The duration between the test and the retest was set to four to seven days to avoid recalling the prior answer and to assume the participant’s health status did not change between the two visits. The ICC2,1 in this study was 0.96, which supports the hypothesis that the PSFS-Ar has high test–retest reliability in the stroke population. Alnahdi et al. [17] translated the PSFS into the Arabic language. They reported very good test–retest reliability of the PSFS-Ar (ICC2,1 = 0.86) in individuals with lower extremity musculoskeletal disorders. Additionally, Stratford et al. reported high reliability (ICC2,1 = 0.97) of the PSFS in participants with mechanical lower back pain [13]. Moreover, the PSFS showed adequate test–retest reliability (ICC2,1 = 0.76) in individuals with different conditions, including neurological and musculoskeletal, among elderly patients with or without cognitive impairments [19]. Additionally, the finding of test–retest reliability of the PSFS was sufficient in the systematic review, which reported that the ICC2,1 value in all conditions was greater than 0.70 [16].

The COSMIN study defined the measurement error as “the systematic and random error of a patient’s score that is not attributed to true changes in the construct to be measured” [51]. The SEM provides insight into the consistency of the repeated responses of an individual over time [52]. Therefore, the amount of error in a measure can be determined using the SEM [52]. The MDC is the smallest change in a measure that is not attributed to random variance [53]. Based on the ICC2,1, the SEM and MDC95 were 0.37 and 1.03, respectively. Therefore, in order to describe a change in a patient functional status as a true change, the PSFS-Ar score is supposed to change by at least 1.03 points. The SEM and MDC90 of the PSFS-Ar have been reported to be 0.64 and 1.49, respectively, in patients with lower extremity musculoskeletal disorders [17]. The SEM of the PSFS for 31 participants in a study on community-dwelling older adults was 1, and the MDC95 was 2.8.78. The SEM and MDC90 for the PSFS in acutely hospitalized patients with different conditions, including neurological and musculoskeletal with and without cognitive impairments were 0.78 and 1.82, respectively [19]. In a systematic review of 57 studies, Pathak et al. [16] reported that the SEM of the PSFS was mostly less than 1, and the MDC values were 1.5 to 3. Moreover, there was no floor and ceiling effect observed in this study, which matches the findings of Alnahdi et al. [2], who reported no floor and ceiling effects for the PSFS-Ar. Similarly, Heldmann et al. [19] demonstrated no floor and ceiling effects.

Construct validity is assessed by testing the hypotheses of direct correlations (negative, positive, or no correlation) between the results of a substantial tool and another outcome measure consistently [54]. For acceptable construct validity, it should include a sample size of at least 50 individuals, and at least 75% of the hypotheses must be satisfied. For standard linear relationship methods, a value near ±1 reflects a significant positive/negative relationship, while a value close to zero indicates no relationship [38,40]. A correlation coefficient that is less than 0.3 indicates a weak relationship. If it is between 0.31 and 0.5, the relationship is moderate, and if it is higher than 0.5, it is strong [38,40]. The BBS, FAC, and SSQOL-Ar (mobility, self-care, and upper limb function) showed a positive moderate correlation with the PSFS-Ar, as stated in the construct validity pre-defined hypotheses. The TUG demonstrated a negative moderate correlation with the PSFS-Ar, which supports the pre-defined hypotheses of the construct validity. Additionally, there was a weak correlation between the SSQOL-Ar (mood) and PSFS-Ar among the pre-defined hypotheses. The pre-defined hypotheses in the study were satisfied by a percentage of 100%, supporting the construct validity of the PSFS-Ar in an individual with stroke. Alnahdi et al. [17] examined the construct validity of the PSFS-Ar for lower extremity musculoskeletal disorders by testing eight pre-defined hypotheses. They reported six satisfied hypotheses out of the eight pre-defined hypotheses (83.3%), which established the construct validity [2]. The two dissatisfied hypotheses were related to pain, which does not measure the same construct as the PSFS-Ar [52]. In another study, the construct validity of the PSFS for upper extremity musculoskeletal disorders showed a moderate correlation when compared with the Upper Extremity Functional Index (UEFI) and Numeric Pain Rating Scale (NPRS) [55]. Pathak et al., reported uncertainty regarding the construct validity of the PSFS that can result from different definitions of construct validity and the level of stringency of the hypotheses tested [16]. This uncertainty was also attributed to the possibility that the reported limited activities in the PSFS are highly specific and mostly affect individuals. Therefore, they reflect low scores of the PSFS, which may alter the correlation with other outcome measures with fixed items [16]. According to a study on the use of the PSFS in patients with ABI, 92% of patients were able to complete the scale and describe the daily activities they could not perform, with the exception of 8% who were unable to express themselves because of severe cognitive or language impairments [21]. Bohannon et al. [20] reported that PSFS was feasible for adult patients with Parkinson’s disease since these individuals could express their disability, including any special activities.

### Limitations and Futures Studies

Patient-specific measurements have the benefit of identifying the most important concerns of each patient. They are more likely to emphasize only the areas that are important to the patients. The lack of standardization of the items in the PSFS might hinder cross-patient comparability. Nonetheless, the PSFS provides unique and individualized assessment for each patient. Although none of the participants in this study showed cognitive impairments based on simple and structured cognitive questions, the lack of standardized cognition measure is one of the limitations. Among the limitations was the fact that most of the participants in this study were male, which limits the generalizability of the result to male individuals with stroke. Future studies should collect more female data for the generalizability of the PSFS-Ar in female patients with stroke. In addition, future studies should explore the responsiveness and minimal clinically important difference for the PSFS-Ar in patients with stroke. While this study emphasizes the benefits of patient-specific measurements and the usefulness of the PSFS-Ar in detecting specific difficulties in functional activities among stroke patients, it does not explicitly address the specific statistical processing methods used or the choice between absolute or relative values. The selection of statistical approaches should consider the research objectives, the nature of the data, and the specific characteristics of the patient population being studied. Both absolute and relative values have their own strengths and limitations, and the appropriate approach should be carefully considered based on the specific context and research goals.

## 5. Conclusions

To the best of our knowledge, this is the first study that assessed PSFS-Ar psychometric properties in the stroke population. The results of the present study showed that the PSFS-Ar has excellent test–retest reliability, acceptable measurement error, and no floor and ceiling effect. We established the construct validity for male individuals with stroke. This study showed that the PSFS-Ar is a self-reported outcome measure that helps to detect specific difficulties with functional activities in patients with stroke. It is easy and understandable for patients to express and report their functional limitations. It might be helpful to pinpoint and rate the disabilities and motivate the patients to reach their own goals.

## Figures and Tables

**Table 1:** Demographic and physical characteristics of the study population.

Variable	Mean (SD) or N (%)
Age	62.3 (11.3)
Gender	
Male	50 (90.9%)
Female	5 (9.1%)
Hemiparetic side	
Right	26 (47.3%)
Left	29 (52.7%)
Type of stroke	
Ischemic	53 (96.4%)
Hemorrhagic	2 (3.6%)
Stroke stages	
Subacute (early/late)	26 (47.3)
Chronic	29 (52.7%)
Diagnosis	
Basal ganglia stroke	15 (27.3%)
Corona radiata stroke	9 (16.4%)
Internal capsule stroke	3 (5.5%)
Corpus callosum stroke	1 (1.8%)
Thalamic stroke	5 (9.1%)
Pontine stroke	8 (14.5%)
Medullary stroke	1 (1.8%)
MCA stroke	7 (12.7%)
PCA stroke	5 (9.1%)
ACA stroke	1 (1.8%)
Dominant hand	
Right hand	51 (92.7%)
Left hand	4 (7.3%)

MCA = middle cerebral artery, PCA = posterior cerebral artery, ACA = anterior cerebral artery.

**Table 2:** Scores of the outcome measures (*n* = 55).

Variable	Median (Range) or N (%)
BBS ^a^	50 (19–56)
TUG ^b^	18 (9–146)
FAC ^c^	
0	0 (0%)
1	8 (14.5%)
2	7 (12.7%)
3	4 (7.3%)
4	13 (23.6%)
5	23 (41.8%)
SSQOL-Ar ^d^	3.3 (1.3–4.9)
EnSQ ^e^	2 (1–5)
FaSQ ^f^	3 (1–5)
LaSQ ^g^	4.6 (1–5)
MoSQ ^h^	2.3 (1–5)
MSQ ^i^	4.2 (1–5)
PeSQ ^j^	3 (1–5)
SCSQ ^k^	3.2 (1–5)
SoSQ ^l^	2 (1–5)
ThSQ ^m^	4 (1–5)
ULFSQ ^n^	3 (1–5)
ViSQ ^o^	4.7 (1–5)
WoSQ ^p^	1 (1–5)

^a^ = Berg Balance Scale, ^b^ = Timed Up And Go, ^c^ = Functional Ambulation Categories, ^d^ = Stroke-Specific Quality of Life Scale, ^e^ = Energy, ^f^ = Family, ^g^ = Language, ^h^ = Mobility, ^i^ = Mood, ^j^ = Personality, ^k^ = Self-care, ^l^ = Social activity, ^m^ = Thinking, ^n^ = Upper limb function ^o^ = Vision, ^p^ = Work.

**Table 3:** PSFS-Ar and GROC results.

Variable	*n* * = 55	*n* ** = 53
PSFS ^a^		
DBT12 ^b^ median (range)	5 (4–12),	5 (4–12)
AT1 ^c^ median (range)	3.8 (0.4–7.4)	3.6 (0.4–7.4)
AT2 ^d^ median (range)	3.4 (0.4–8)	3.4 (0.4–8)
GROC ^e^ median (range)	0 (−2–4)	0 (−2–2)

^a^ = Patient-Specific Functional Scale, ^b^ = days between time 1 and time 2 of PSFS, ^c^ = average of time 1 of PSFS, ^d^ = average of time 2 of PSFS, ^e^ = Global Rating of Change, * = total number of patients, ** = two patients scored 3 and 4 in the GROC were excluded from test–retest.

**Table 4:** Test–retest data, measurement error (*n* = 53).

	Mean (SD)	Mean Difference (95%CI)	ICC_2,1_ ^a^ (95%CI)	SEM ^b^	MDC_95_ ^c^
Test	3.5 (±1.8)	−0.1 (−0.21–0.05)	0.96 ^1^ (0.94–0.98) *p* < 0.001	0.37 ^2^	1.03 ^3^
Retest	3.6 (±1.8)				

^a^ = Intraclass correlation coefficient (two-way random model for agreement), ^b^ = standard error of measurement, ^c^ = minimal detectable change with 95% confidence, ^1^ = ICC_2,1_ showed excellent test–retest, ^2^ = SEM = 1.84 × √(1 − ICC_2,1_), ^3^ = MDC_95_ = SEM × 1.96 × √2.

**Table 5:** Correlation between PSFS-Ar and other outcome measures. (*n* = 55).

Variable	*R*	*p*
BBS ^a,^*	0.45	*p* < 0.001
TUG ^b,^*	−0.39	*p* = 0.004
FAC ^c,^*	0.47	*p* < 0.001
SSQOL-Ar ^d^		
Mobility domain *	0.49	*p* < 0.001
Mood domain *	0.16	*p* = 0.2
Self-care domain *	0.5	*p* < 0.001
Upper limb function domin *	0.43	*p* = 0.001

^a^ = Berg Balance Scale, ^b^ = Timed Up and Go, ^c^ = Functional Ambulation Categories, ^d^ = Stroke-Specific Quality of Life, * = Spearman.

## Data Availability

Data are available from the first author (MAA). The data will be provided on request.
